# Inhibitory Effects of Glycyrrhetinic Acid on DNA Polymerase and Inflammatory Activities

**DOI:** 10.1155/2012/650514

**Published:** 2011-07-14

**Authors:** Tsukasa Ishida, Yoshiyuki Mizushina, Saori Yagi, Yasuhiro Irino, Shin Nishiumi, Ikuya Miki, Yasuyuki Kondo, Shigeto Mizuno, Hiromi Yoshida, Takeshi Azuma, Masaru Yoshida

**Affiliations:** ^1^Department of Medical Pharmaceutics, Kobe Pharmaceutical University, Higashinada-ku, Kobe 658-8558, Japan; ^2^Laboratory of Food & Nutritional Sciences, Department of Nutritional Science, Kobe-Gakuin University, Nishi-ku, Kobe, Hyogo 651-2180, Japan; ^3^Cooperative Research Center of Life Sciences, Kobe-Gakuin University, Chuo-ku, Kobe, Hyogo 650-8586, Japan; ^4^The Integrated Center for Mass Spectrometry, Kobe University Graduate School of Medicine, Chuo-ku, Kobe, Hyogo 650-0017, Japan; ^5^Division of Gastroenterology, Department of Internal Medicine, Kobe University Graduate School of Medicine, Chuo-ku, Kobe, Hyogo 650-0017, Japan; ^6^Division of Metabolomics Research, Kobe University Graduate School of Medicine, Chuo-ku, Kobe, Hyogo 650-0017, Japan

## Abstract

We investigated the inhibitory effect of three glycyrrhizin derivatives, such as Glycyrrhizin (compound **1**), dipotassium glycyrrhizate (compound **2**) and glycyrrhetinic acid (compound **3**), on the activity of mammalian pols. Among these derivatives, compound **3** was the strongest inhibitor of mammalian pols **α**, **β**, **κ**, and **λ**, which belong to the B, A, Y, and X families of pols, respectively, whereas compounds **1** and **2** showed moderate inhibition. Among the these derivatives tested, compound **3** displayed strongest suppression of the production of tumor necrosis factor-**α** (TNF-**α**) induced by lipopolysaccharide (LPS) in a cell-culture system using mouse macrophages RAW264.7 and peritoneal macrophages derived from mice. Moreover, compound **3** was found to inhibit the action of nuclear factor-**κ**B (NF-**κ**B) in engineered human embryonic kidney (HEK) 293 cells. In addition, compound **3** caused greater reduction of 12-*O*-tetradecanoylphorbol-13-acetate-(TPA-) induced acute inflammation in mouse ear than compounds **1** and **2**. In conclusion, this study has identified compound **3**, which is the aglycone of compounds **1** and **2**, as a promising anti-inflammatory candidate based on mammalian pol inhibition.

## 1. Introduction

The human genome encodes at least 15 DNA polymerases (pols) that conduct cellular DNA synthesis [1, 2]. Eukaryotic cells contain 3 replicative pols (*α*, *δ*, and *ε*), 1 mitochondrial pol (*γ*), and at least 11 nonreplicative pols [*β*, *ζ*, *η*, *θ*, *ι*, *κ*, *λ*, *μ*, *ν*, terminal deoxynucleotidyl transferase (TdT) and REV1] [3, 4]. Pols have a highly conserved structure, which means that their overall catalytic subunits show little variance among species. Enzymes with conserved structures usually perform important cellular functions, the maintenance of which provides evolutionary advantages. On the basis of sequence homology, eukaryotic pols can be divided into 4 main families, termed A, B, X, and Y [4]. Family A includes mitochondrial pol *γ*, as well as pols *θ* and *ν*. Family B includes 3 replicative pols (*α*, *δ*, and *ε*) and pol *ζ*. Family X comprises pols *β*, *λ*, and *μ*, as well as TdT, and lastly, family Y includes pols *η*, *ι*, and *κ*, in addition to REV1.

We have been screening for selective inhibitors of each pol derived from natural products, in particular from traditional medical plants, food materials and food additives, for more than 15 years [5, 6]. Licorice (fabaceae and glycyrrhiza), a well-known herb plant with biological properties, has been widely used in food additives (sweetener and flavoring agent), nutraceuticals (liver protection), and the treatment of various inflammatory diseases since ancient times [7]. Licorice root has been used since ancient Egyptian, Greek, and Roman times in the West, and since the Former Han era (the 2nd-3rd century B.C.) in ancient China in the East. In traditional Chinese medicine, licorice is one of the most frequently used drugs. There are many reports about its pharmacological actions and physiological functions, such as detoxification, induction of decreased blood glucose, and antitumor, anti-inflammatory, hypocholesterolemic, antiestrogen, antihistamine, antiallergic hepatitis, antileukemia, anticancer, and antibiotic-like effects [8]. 

**Figure 1:**
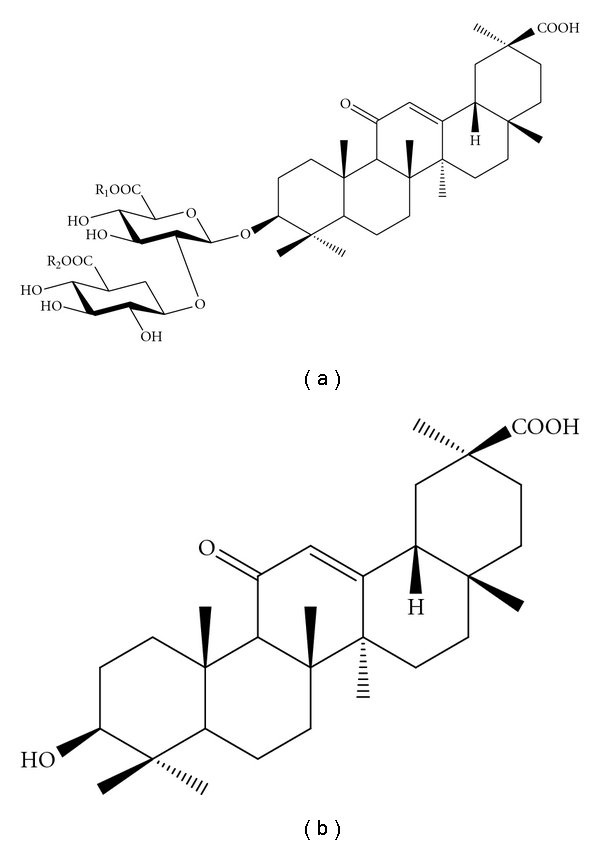
Structure of the glycyrrhizin derivatives. (a) Glycyrrhizin (3-*O*-(2-*O*-*β*-D-glucopyranuronosyl-*α*-D-glucopyranuronosyl)-18*β*-glycyrrhetinic acid; compound **1: **R_1_ and R_2_ = H) and dipotassium glycyrrhizate (compound **2**: R_1_ and R_2_ = K^+^). (b) Glycyrrhetinic acid (compound **3**).

The seed stock of licorice (*Glycyrrhiza glabra* L.) has 6 varieties, and licorice production is widely distributed over the Eurasian continent, South Europe, Central Asia, China and Russia. In particular, *G. inflata*, *G. uralensis,* and *G. glabra* are utilized in the production of sweeteners, cosmetics, and medicines. The major constituents of licorice root are triterpenoid saponins, such as glycyrrhizin (3–8%). Glycyrrhizin (glycyrrhizic acid and glycyrrhizinic acid) is a sweet-tasting compound, and is 30–50 times as sweet as sucrose. Glycyrrhizin (compound **1**) consists of one molecule of glycyrrhetinic acid (compound **3**) and two molecules of glucuronic acid (Figure 1), and it is converted to these constituents by acidic hydrolysis [9]. 

In our studies of pol inhibitors, we have found that pol *λ*-selective inhibitors, such as curcumin derivatives [10–12], have anti-inflammatory activity against 12-*O*-tetradecanoylphorbol-13-acetate-(TPA-) induced inflammation [13–15]. Although tumor promoters, such as TPA, are classified as compounds that promote tumor formation [16], they also cause inflammation and are commonly used as artificial inducers of inflammation in order to screen for anti-inflammatory agents [17]. Tumor promoter-induced inflammation can be distinguished from acute inflammation, which is exudative and accompanied by fibroblast proliferation and granulation. The tumor promoter TPA is frequently used to search for new types of anti-inflammatory compound. TPA not only causes inflammation, but also influences mammalian cell growth [18], suggesting that the molecular basis of the inflammation stems from pol reactions related to cell proliferation. This relationship, however, needs to be investigated more closely. 

In this study, we have investigated the inhibitory effects of glycyrrhizin (compound **1**) and its derivatives, including the potassium salt of compound **1** (dipotassium glycyrrhizate, compound **2**) and glycyrrhetinic acid (compound **3**) (Figure 1), on mammalian pol activity and inflammatory responses both *in vitro* and *in vivo*. In particular, we demonstrate that these compounds exert inhibitory effects against TNF-*α* production and NF-*κ*B activation in cell culture models of inflammatory response. The relationship between the inhibition of pols and the anti-inflammatory action of the glycyrrhizin derivatives is discussed.

## 2. Materials and Methods

### 2.1. Materials

Glycyrrhizin (3-*O*-(2-*O*-*β*-D-glucopyranuronosyl-*α*-D-glucopyranuronosyl)-18*β*-glycyrrhetinic acid, compound **1**), dipotassium glycyrrhizate (compound **2**) and glycyrrhetinic acid (compound **3**) were obtained from Maruzen Pharmaceuticals Co., Ltd. (Onomichi, Hiroshima, Japan), and each compound was purified to special grade purity. These structures are shown in Figure 1. Chemically synthesized DNA templates, such as poly(dA), was purchased from GE Healthcare Bio-Sciences (Little Chalfont, UK). Radioisotope-labeled nucleotides, such as [^3^H]-deoxythymidine 5′-triphosphate (dTTP) (43 Ci/mmol), was purchased from MP Biomedicals, LLC (Tokyo, Japan). The oligo(dT)_18_ DNA primer was customized by Sigma-Aldrich Japan K.K. (Hokkaido, Japan). Lipopolysaccharide (LPS) and TPA were purchased from Sigma-Aldrich. All other reagents were of analytical grade and were purchased from Nacalai Tesque Inc. (Kyoto, Japan).

### 2.2. Mammalian Pol Assays

Pol *α* was purified from calf thymus by immunoaffinity column chromatography as described by Tamai et al. [19]. The human pol *γ* catalytic gene was cloned into pFastBac (Invitrogen Japan K.K., Tokyo Japan). Histidine-tagged enzyme was expressed using the BAC-TO-BAC HT Baculovirus Expression System according to the supplier's manual (Life Technologies, MD) and purified using ProBound resin (Invitrogen Japan K.K.) [20]. A truncated form of pol *κ* (residues 1–560) with a 6xHis tag attached at the C-terminus was overproduced in *E. coli* and purified as described previously [21]. Recombinant human His-pol *λ* was overexpressed and purified according to a method described previously [22]. 

The reaction mixture for calf pol *α* was described previously [23, 24]. The reaction mixture for human pol *γ* was previously described by Umeda et al. [20]. The reaction mixtures for mammalian pols *κ* and *λ* were the same as that for calf pol *α*. The components of the pol assay were poly(dA)/oligo(dT)_18_ (A/T = 2/1) and dTTP as the DNA template-primer and 2′-deoxynucleoside 5′-triphosphate (dNTP) substrate, respectively. The glycyrrhizin derivatives (i.e., compounds **1**–**3**) were dissolved in distilled dimethyl sulfoxide (DMSO) at various concentrations and sonicated for 30 sec. The sonicated samples (4 *μ*L) were mixed with 16 *μ*L of each enzyme (final amount, 0.05 units) in 50 mM Tris-HCl (pH 7.5) containing 1 mM dithiothreitol, 50% glycerol, and 0.1 mM EDTA, and kept at 0°C for 10 min. These inhibitor enzyme mixtures (8 *μ*L) were added to 16 *μ*L of each of standard enzyme reaction mixture (50 mM Tris-HCl [pH 7.5], 1 mM dithiothreitol, 1 mM MgCl_2_, 15% glycerol, 10 *μ*M poly(dA)/oligo(dT)_18_, and 10 *μ*M [^3^H]-dTTP), and incubation was carried out at 37°C for 60 min. Activity in the absence of inhibitor was considered to be 100%, and the activity remaining at each concentration of inhibitor was determined relative to this value. One unit of pol activity was defined as the amount of enzyme that catalyzed the incorporation of 1 nmol dNTP (dTTP) into the synthetic DNA template-primer (poly(dA)/oligo(dT)_18_, A/T = 2/1) in 60 min at 37°C under normal reaction conditions for each enzyme (scintillation counts: approximately 1 pmol of incorporated radioactive nucleotide = 100 cpm) [23, 24].

### 2.3. Animal Experiments

All animal studies were performed according to the guidelines outlined in the “Care and Use of Laboratory Animals” of Kobe University. The animals were anesthetized with pentobarbital before undergoing cervical dislocation. Female 8-week-old C57BL/6 mice that had been bred in-house with free access to food and water were used for all experiments. All of the mice were maintained under a 12-h light/dark cycle and housed at a room temperature of 25°C.

### 2.4. Cell Culture

A mouse macrophage cell line, RAW264.7, was obtained from American Type Culture Collection (ATCC) (Manassas, Va, USA). The cells were cultured in Eagle's Minimum Essential Medium (MEM) supplemented with 4.5 g of glucose per liter plus 10% fetal calf serum, 5 mM L-glutamine, 50 units/mL penicillin and 50 units/mL streptomycin. HEK-Blue hTLR4 cells were purchased from InvivoGen (San Diego, Calif, USA), and were maintained in complete Dulbecco minimal essential medium with selective antibiotics, in accordance with the manufacturer's instructions (InvivoGen). The cells were cultured at 37°C in standard medium in a humidified atmosphere of 5% CO_2_–95% air.

### 2.5. Preparation of Peritoneal Macrophages

Female C57BL/6 mice were injected intraperitoneally with phosphate-buffered saline (PBS), and the peritoneal cavity of the mice was washed with PBS. The PBS was collected, and peritoneal macrophages were separated from the PBS by centrifugation at 1,500 ×g for 10 min.

### 2.6. Measurement of Cytotoxicity on a Cell-Culture Medium

Approximately 1 × 10^4^ cells per well were inoculated into 96-well microplates, and then compounds **1**–**3** were diluted to various concentrations and applied to each well. After incubation for 24 h, the survival rate of RAW264_*·*_7 cells was determined by MTT (3-[4,5-dimethylthiazol-2-yl]-2,5-diphenyltetrazolium bromide) assay [25].

### 2.7. Measurement of TNF-*α* in a Cell-Culture Medium

RAW264_*·*_7 cells or peritoneal macrophages were placed in a 12-well plate at 5 × 10^4^ cells/well and incubated for 24 h. The cells were pretreated with various concentrations of compounds **1**–**3** for 30 min before the addition of 100 ng/mL LPS. After stimulation with LPS for 24 h, the cell culture medium was collected to measure the amount of TNF-*α* secreted. The concentration of TNF-*α* in the culture medium was quantified by using a commercially available enzyme-linked immunosorbent assay (ELISA) development system (Bay Bioscience Co., Ltd., Kobe, Japan) in accordance with the manufacturer's protocol.

### 2.8. Measurement of the Nuclear Translocation of NF-*κ*B in HEK293 Cells

HEK-Blue hTLR4 cells are engineered human embryonic kidney (HEK) 293 cells that stably coexpress human Toll-like receptor 4 (TLR4) and an NF-*κ*B-inducible secreted embryonic alkaline phosphatase (SEAP) reporter gene. These cells were placed in a 96-well plate at 1 × 10^4^ cells/well and incubated for 24 h. The cells were pre-incubated with various concentrations of compounds **1**–**3** for 30 min before the addition of 1 ng/mL LPS. After stimulation with LPS for 24 h, NF-*κ*B-induced SEAP activity was assessed by using QUANTI-Blue (a medium used for the detection and quantification of SEAP; InvivoGen) and by reading the absorbance at 650 nm by means of an ELISA plate reader.

### 2.9. TPA-Induced Anti-Inflammatory Assay in Mouse

The mouse inflammatory test was performed according to Gschwendt's method [26]. In brief, an acetone solution of compounds **1**–**3** (250 or 500 *μ*g in 20 *μ*L) or 20 *μ*L of acetone as a vehicle control was applied to the inner part of the mouse ear. Thirty minutes after the test compound was applied, a TPA solution (0.5 *μ*g/20 *μ*L of acetone) was applied to the same part of the ear. To the other ear of the same mouse, methanol, followed by TPA solution, was applied as a control. After 7 h, a disk (6 mm diameter) was obtained from each ear and weighed. The inhibitory effect (IE) is presented as a ratio of the increase in weight of the ear disks: IE: {[(TPA only) – (tested compound plus TPA)]/[(TPA only) – (vehicle)] × 100}.

## 3. Results

### 3.1. Effect of Glycyrrhizin Derivatives (Compounds **1**–**3**) on Mammalian Pol Activity

**Figure 2:**
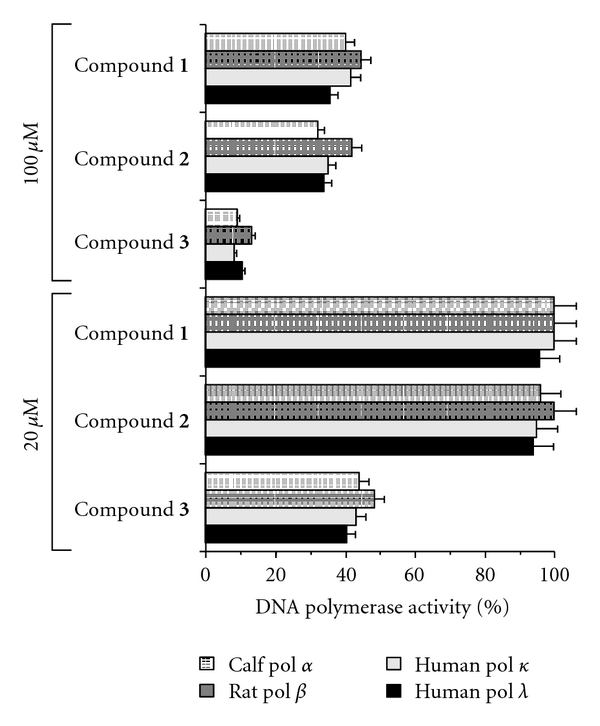
Inhibitory effects of glycyrrhizin derivatives (compounds **1**–**3**) on the activity of mammalian pols. Each compound (20 and 100 *μ*M) was incubated with calf pol *α* (B-family pol), human pol *γ* (A-family pol), human pol *κ* (Y-family pol), and human pol *λ* (X-family pol) (0.05 units each). Pol activity in the absence of the compound was taken as 100%, and the relative activity is shown. Data are shown as the mean ± SE (*n* = 4).

Initially, we investigated the *in vitro* biochemical action of glycyrrhizin (compound **1**) and its derivatives (compounds **2** and **3**). The inhibition of four mammalian pols, namely, calf pol *α*, human pol *γ*, human pol *κ*, and human pol *λ*, by each compound at 20 and 100 *μ*M was investigated. Pols *α*, *γ*, *κ*, and *λ* were used as representatives of the B, A, Y, and X families of pols, respectively [1–3]. As shown in Figure 2, at 100 *μ*M these compounds inhibited the activity of all mammalian pols tested, because the relative pol activity was less than 50% after incubation with these compounds. At 20 *μ*M, compound **3 **also inhibited the activity of these pols, whereas compounds **1** and **2** had no effect; therefore, the inhibitory effect of these compounds on mammalian pols was ranked as follows: compound **3** > compound **1** = compound **2**. Compound **3** showed almost the same strength of inhibition among the four mammalian pols tested; that is, the concentration of compound **3** giving 50% inhibition of pols *α*, *γ*, *κ*, and *λ* was 16.1, 19.3, 15.8, and 13.7 *μ*M, respectively. When activated DNA (i.e., bovine deoxyribonuclease I-treated DNA) and dNTP were used as the DNA template-primer and nucleotide substrate instead of synthesized DNA [poly(dA)/oligo(dT)_18_ (A/T = 2/1)] and dTTP, respectively, the inhibitory effects of these compounds did not change (data not shown).

### 3.2. Inhibitory Effect of Glycyrrhizin Derivatives (Compounds **1**–**3**) on LPS-Induced Inflammatory Responses in Cultured Cells

**Figure 3:**
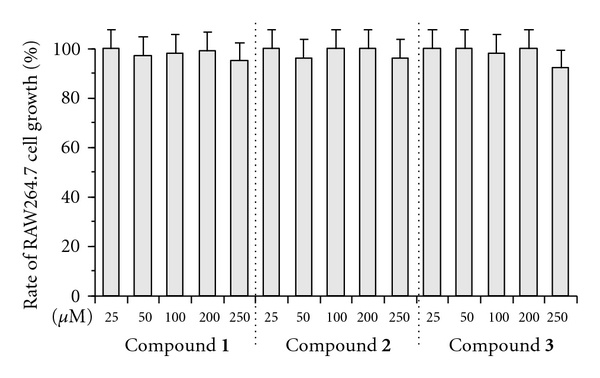
Effect of glycyrrhizin derivatives (compounds **1**–**3**) on the proliferation of the mouse macrophage RAW264.7 cell growth. The cells were added the indicated concentrations of each compound and incubated for 24 h, and the rate of cultured cell growth inhibition was determined by MTT assay [25]. Cell growth inhibition of the cells in the absence of the compound was taken as 100%. Data are shown as the mean ± SE (*n* = 5).

**Figure 4:**
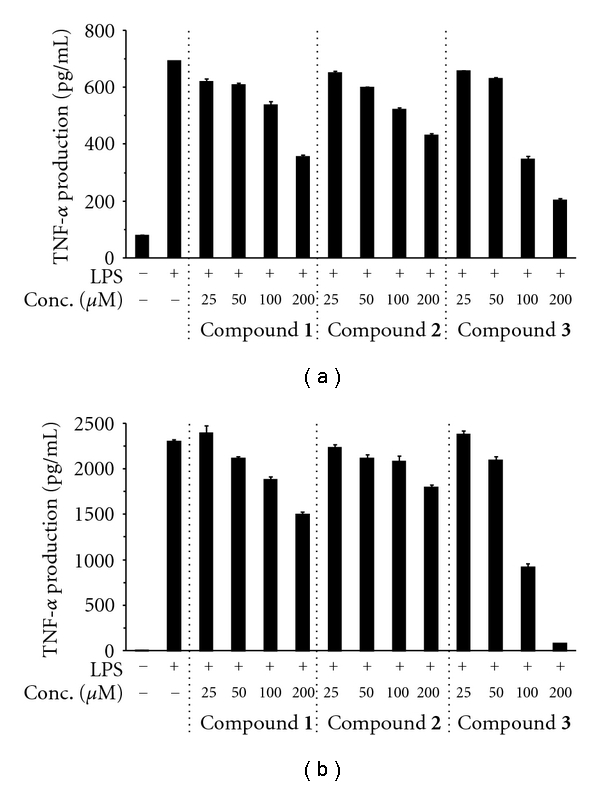
Inhibitory effects of glycyrrhizin derivatives (compounds **1**–**3**) on LPS-induced production of TNF-*α* in mouse macrophages. (a) The mouse macrophage cell line RAW264.7 was pretreated with the indicated concentrations of the glycyrrhizin derivatives for 30 min, and then treated with 100 ng/mL LPS for 24 h. (b) Peritoneal macrophages derived from mice were pretreated with the indicated concentrations of each compound for 30 min, and then with 100 ng/mL LPS for 24 h. The TNF-*α* concentration in the cell medium was measured by ELISA. Data are shown as the mean ± SE (*n* = 5).

Next, we investigated whether the three glycyrrhizin derivatives could inhibit both the reduction of TNF-*α* production and the nuclear translocation of NF-*κ*B caused by LPS stimulation in cultured cells. The inflammatory cytokine TNF-*α* activates the NF-*κ*B signaling pathway by binding to the TNF-*α* receptor (TNFR) and thereby initiates an inflammatory response, resulting in various inflammatory diseases [27]. In cultured macrophage RAW264.7 cells, no compound showed cytotoxicity at 25 to 250 *μ*M (Figure 3); therefore, the LD_50_ values of compounds **1**–**3** were >250 *μ*M. These compounds also had no effect on the cell proliferation of peritoneal macrophages (data not shown). As shown in Figure 4(a), RAW264.7 cells produced 693 pg/mL of TNF-*α* after LPS treatment. Compounds **1–3 **slightly suppressed this LPS-stimulated production of TNF-*α*, showing dose-dependent inhibition at 100 and 200 *μ*M. The suppression of TNF-*α* production by compound** 3 **was stronger than that by compounds **1** and **2**.  Figure 4(b) shows the dose-dependent suppression of LPS-evoked TNF-*α* production in peritoneal macrophages derived from mice by the glycyrrhizin derivatives. The inhibitory effect of compounds **1**–**3** showed almost the same tendency in peritoneal macrophages as in the macrophage cell line RAW264.7 although compound **3** significantly suppressed the production of TNF-*α* in peritoneal macrophages. From these results, the strength of the inhibitory effect of these compounds can be ranked as follows: compound **3** > compound **1** = compound **2**.

**Figure 5:**
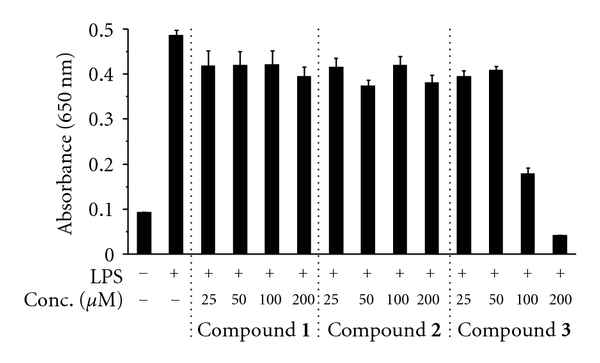
Inhibitory effects of glycyrrhizin derivatives (compounds **1**–**3**) on nuclear translocation of NF-*κ*B in engineered HEK293 cells. HEK-Blue hTLR4 cells were pretreated for 30 min with the indicated concentrations of the glycyrrhizin derivatives and then treated with 1 ng/mL LPS for 24 h. NF-*κ*B-induced SEAP activity was assessed by using QUANTI-Blue and by reading the absorbance at 650 nm via an ELISA plate reader. Data are shown as the mean ± SE (*n* = 5).

NF-*κ*B is known to be the rate-controlling factor in inflammatory responses. We, therefore, examined the inhibitory effect of compounds **1**–**3** on the LPS-induced nuclear translocation of NF-*κ*B in RAW264.7 cells (Figure 5). In this experiment, we used HEK-Blue hTLR4 cells, which are HEK293 cells that have been engineered to report TLR4-linked NF-*κ*B activation. In brief, this cell line is transfected with TLR4 and an NF-*κ*B-inducible alkaline phosphatase reporter gene system. On interaction with the appropriate ligand, TLR4 transduces a signal that results in NF-*κ*B activation. In this assay system, the amount of NF-*κ*B undergoing nuclear translocation in the cells after LPS stimulation was significantly reduced by compound **3** at 100 and 200 *μ*M. By contrast, compounds **1** and **2** had no effect on the LPS-stimulated nuclear translocation of NF-*κ*B; thus, the ranking was compound **3**  ≫ compound **1** = compound **2**. These results demonstrate that compound **3** can strongly suppress the nuclear translocation of NF-*κ*B by inhibiting the production of TNF-*α*. The effects of glycyrrhizin derivatives on the molecular mechanism underlying inflammatory responses will be addressed in future studies.

### 3.3. Effect of Glycyrrhizin Derivatives (Compounds **1**–**3**) on TPA-Induced Anti-Inflammatory Activity

**Figure 6:**
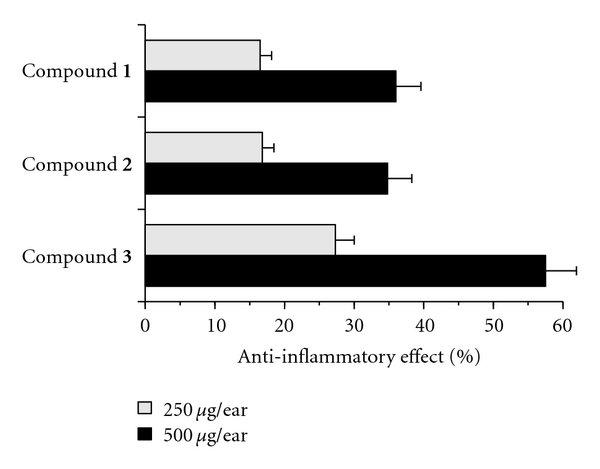
Anti-inflammatory activity of glycyrrhizin derivatives toward TPA-induced edema on mouse ear. Each compound (250 *μ*g, gray bar; and 500 *μ*g, black bar) was applied individually to one ear of a mouse, and after 30 min TPA (0.5 *μ*g) was applied to both ears. Edema was evaluated after 7 h. The inhibitory effect is expressed as a percentage of edema. Data are shown as the means ± SE (*n* = 6).

In a previous study of pol inhibitors, we found that there is a relationship between pol *λ* inhibitors and TPA-induced acute anti-inflammatory activity [6, 13, 14]. Thus, using the mouse ear inflammatory test, we examined the anti-inflammatory activity of the glycyrrhizin derivatives. Application of TPA (0.5 *μ*g) to the mouse ear induced edema, resulting in a 241% increase in the weight of the ear disk 7 h after application. As shown in Figure 6, pretreatment with compounds **1**–**3** dose-dependently suppressed inflammation, and the effect of these compounds was ranked as follows: compound **3** > compound **1** = compound **2**. Thus, these *in vivo* data from the mouse ear study showed almost the same trend as the LPS-induced inflammatory response data from cultured cells (Figure 5). Furthermore, the anti-inflammatory effect of these compounds showed the same tendency as their inhibitory effect on mammalian pols including pol *λ*, which was strongly inhibited by compound **3** (Figure 2). These results suggest that inhibition of pol *λ* inhibitory activity has a positive correlation with the anti-inflammatory activity observed.

## 4. Discussion

We have shown here that glycyrrhetinic acid (compound **3**) was the strongest inhibitor of mammalian pols *α*, *γ*, *κ*, and *λ* (Figure 2), and this compound prevented the inflammatory response among the three glycyrrhizin derivatives (compounds **1**–**3**) tested (Figures 4 to 6). Compound **3** is the aglycone of compound **1**, and the pentacyclic triterpenoid structure must be important for the inhibitory activity shown by these compounds. Glycyrrhizin (compounds **1**) and dipotassium glycyrrhizate (compound **2**), which is the dipotassium salt of compound **1**, are triterpenoid saponin glycosides, and they both have two molecules of glucuronic acid (Figure 1). Compound **2** is a major sweet-tasting food additive, and it is 5-fold sweeter than compound **1** [7]. The inhibitory effect of compound **1** on pol activity and inflammation showed the same tendency as that of compound **2**; therefore, the salt form had no effect on the inhibitory activities of glycyrrhizin. 

Eukaryotic cells reportedly contain 15 pol species belonging to four families: namely, family A (pols *γ*, *θ*, and *ν*), family B (pols *α*, *δ*, *ε*, and *ζ*), family X (pols *β*, *λ*, and *μ* and TdT) and family Y (pols *η*, *ι*, and *κ* and REV1) [3, 4]. As reported previously, the phenolic compound curcumin, which is a known anti-inflammatory agent, is a pol *λ*-specific inhibitor [6, 13, 14]. Intriguingly, on the basis of compound **3**, the principle molecular target of the glycyrrhizin derivatives is also pol *λ*. Among the X family of pols, pol *λ* has an unclear biochemical function, although it seems to work in a similar way to pol *β* [28]. Pol *β* is involved in the short-patch base excision repair (BER) pathway [29–32], as well as playing an essential role in neural development [33]. Recently, pol *λ* was found to possess 5′-deoxyribose-5-phosphate (dRP) lyase activity, but no apurinic/apyrimidinic (AP) lyase activity [34]. Pol *λ* is able to substitute for pol *β* during *in vitro *BER, suggesting that pol *λ* also participates in BER. Northern blot analysis indicated that transcripts of pol *β* are abundantly expressed in the testis, thymus, and brain in rats [35], whereas pol *λ* is efficiently transcribed mostly in the testis [36]. Bertocci et al. reported that mice in which pol *λ* expression is knocked out are not only viable and fertile, but also display a normal hypermutation pattern [37]. 

As well as causing inflammation, TPA influences cell proliferation and has physiological effects on cells owing to its tumor-promoting activity [18]. Therefore, anti-inflammatory agents are expected to suppress DNA replication/repair/recombination in nuclei in relation to the action of TPA. Because pol *λ* is a repair/recombination-related pol [28], our finding—that pol *λ* is the molecular target of glycyrrhizin derivatives—is in good agreement with this expected mechanism of anti-inflammatory agents. As a result, any pol *λ* inhibitor might also be an inhibitor of inflammation. 

Compound **1** has been reported to possess various pharmacological properties such as anti-inflammatory activity [38], inhibition of prostaglandin E2 production in rat macrophages [39], antiallergic activity [40], antiviral activity [41, 42], and induction of interferon-*γ* [43]. In Japan, a preparation of compound **1**, Stronger-Neo Minophagen C, has been used extensively to treat chronic hepatitis for more than 30 years. Compound **3** is also known to have wide pharmacological effects such as anti-inflammatory [44, 45], antitumor [46], and antihepatotoxic [47] activities, and inhibition of the growth of mouse melanoma [48]. In 1989, it was reported that compound **3** strongly inhibits renal 11*β*-hydroxysteroid dehydrogenase in rat [49]; this inhibition has been regarded as a cause of the pseudoaldosteronism that is occasionally induced by the administration of a compound **3** preparation or Carbenoxolone. However, the mechanisms underlying the therapeutic effects of the glycyrrhizin derivatives remain unknown. 

In this study, therefore, we investigated the inhibitory effect of the glycyrrhizin derivatives on mammalian pols, which are responsible for DNA replication leading to cell proliferation and DNA repair/recombination, as well as the relationship between the degree of the suppression of LPS-evoked TNF-*α* production and anti-inflammatory activity. The molecular mechanism that links the LPS-induced inflammatory response and anti-inflammatory activity in the model of TPA-induced ear edema is unknown. Because activated NF-*κ*B has been observed in a model of TPA-induced ear edema [50], the anti-inflammatory effects of compound **3** may be, at least in part, dependent on the inhibition of NF-*κ*B activation. Our study indicates that compound **3** is useful as an NF-*κ*B inhibitor and may be a potent chemopreventive agent against inflammation. As a result, we found a positive correlation between the pol inhibitory activity and anti-inflammatory activity of compound **3**. The relationship between these activities, namely, pol *λ* inhibition and anti-inflammatory action, may be useful as a new and convenient *in vitro* assay to screen for novel anti-inflammatory compounds.

## 5. Conclusions

Our study is the first to demonstrate that glycyrrhetinic acid (compound **3**), which is the aglycone of glycyrrhizin (compounds **1**), potently inhibited the activity of mammalian pols including pol *λ*. Compound **3** also reduced TNF-*α* production and NF-*κ*B activation and suppressed mouse ear inflammation stimulated by TPA. Thus, compound **3** could be an anti-inflammatory agent based on pol *λ* inhibition.

## Abbreviations

pol: DNA polymerase (E.C. 2.7.7.7)
TPA: 12-*O*-tetradecanoylphorbol-13-acetate
TNF: Tumor necrosis factor
NF: Nuclear factor
LPS: Lipopolysaccharide
TdT: Terminal deoxynucleotidyl transferase
dTTP: 2′-deoxythymidine 5′-triphosphate
DMSO: Dimethylsulfoxide
PBS: Phosphate buffered saline
ELISA: Enzyme-linked immunosorbent assay
HEK: Human embryonic kidney
TRL: Toll-like receptor
SEAP: Secreted embryonic alkaline phosphatase
IE: Inhibitory effect
BER: Base excision repair
dRP: 5′-deoxyribose-5-phosphate
AP: Apurinic/apyrimidinic.

## References

[1] Hübscher U, Maga G, Spadari S (2002). Eukaryotic DNA polymerases. *Annual Review of Biochemistry*.

[2] Bebenek K, Kunkel TA, Yang W (2004). DNA repair and replication. *Advances in Protein Chemistry*.

[3] Takata KI, Shimizu T, Iwai S, Wood RD (2006). Human DNA polymerase N (POLN) is a low fidelity enzyme capable of error-free bypass of 5S-thymine glycol. *Journal of Biological Chemistry*.

[4] Friedberg EC, Feaver WJ, Gerlach VL (2000). The many faces of DNA polymerases: strategies for mutagenesis and for mutational avoidance. *Proceedings of the National Academy of Sciences of the United States of America*.

[5] Sakaguchi K, Sugawara F, Mizushina Y (2002). Inhibitors of eukaryotic DNA polymerases. *Seikagaku*.

[6] Mizushina Y (2009). Specific inhibitors of mammalian DNA polymerase species. *Bioscience, Biotechnology and Biochemistry*.

[7] Eisenbrand G (2006). Glycyrrhizin: opinion of the Senate Commission on Food Safety (SKLM) of the German Research Foundation (DFG). *Molecular Nutrition and Food Research*.

[8] Shibata S (2000). A drug over the millennia: pharmacognosy, chemistry, and pharmacology of licorice. *Yakugaku Zasshi*.

[9] Lin G, Nnane IP, Cheng TY (1999). The effects of pretreatment with glycyrrhizin and glycyrrhetinic acid on the retrorsine-induced hepatotoxicity in rats. *Toxicon*.

[10] Mizushina Y, Kamisuki S, Kasai N (2002). Petasiphenol: a DNA polymerase *λ* inhibitor. *Biochemistry*.

[11] Mizushina Y, Ishidoh T, Takeuchi T (2005). Monoacetylcurcumin: a new inhibitor of eukaryotic DNA polymerase *λ* and a new ligand for inhibitor-affinity chromatography. *Biochemical and Biophysical Research Communications*.

[12] Takeuchi T, Ishidoh T, Iijima H (2006). Structural relationship of curcumin derivatives binding to the BRCT domain of human DNA polymerase *λ*. *Genes to Cells*.

[13] Mizushina Y, Hirota M, Murakami C (2003). Some anti-chronic inflammatory compounds are DNA polymerase *λ*-specific inhibitors. *Biochemical Pharmacology*.

[14] Mizushina Y, Takeuchi T, Kuramochi K (2007). Study on the molecular structure and bio-activity (DNA polymerase inhibitory activity, anti-inflammatory activity and anti-oxidant activity) relationship of curcumin derivatives. *Current Bioactive Compounds*.

[15] Nishida M, Nishiumi S, Mizushina Y (2010). Monoacetylcurcumin strongly regulates inflammatory responses through inhibition of NF-*κ*B activation. *International Journal of Molecular Medicine*.

[16] Hecker E (1978). *Carcinogenesis*.

[17] Fujiki H, Sugimura T (1987). *Advances in Cancer Research*.

[18] Nakamura Y, Murakami A, Ohto Y, Torikai K, Tanaka T, Ohigashi H (1995). Suppression of tumor promoter-induced oxidative stress and inflammatory responses in mouse skin by a superoxide generation inhibitor 1’- acetoxychavicol acetate. *Cancer Research*.

[19] Tamai K, Kojima K, Hanaichi T (1988). Structural study of immunoaffinity-purified DNA polymerase *α*-DNA primase complex from calf thymus. *Biochimica et Biophysica Acta*.

[20] Umeda S, Muta T, Ohsato T, Takamatsu C, Hamasaki N, Kang D (2000). The D-loop structure of human mtDNA is destabilized directly by 1- methyl-4-phenylpyridinium ion (MPP+), a parkinsonism-causing toxin. *European Journal of Biochemistry*.

[21] Ohashi E, Murakumo Y, Kanjo N (2004). Interaction of hREV1 with three human Y-family DNA polymerases. *Genes to Cells*.

[22] Shimazaki N, Yoshida K, Kobayashi T, Toji S, Tamai K, Koiwai O (2002). Over-expression of human DNA polymerase *λ* in *E. coli* and characterization of the recombinant enzyme. *Genes to Cells*.

[23] Mizushina Y, Tanaka N, Yagi H (1996). Fatty acids selectively inhibit eukaryotic DNA polymerase activities in vitro. *Biochimica et Biophysica Acta*.

[24] Mizushina Y, Yoshida S, Matsukage A, Sakaguchi K (1997). The inhibitory action of fatty acids on DNA polymerase. *Biochimica et Biophysica Acta*.

[25] Mosmann T (1983). Rapid colorimetric assay for cellular growth and survival: application to proliferation and cytotoxicity assays. *Journal of Immunological Methods*.

[26] Gschwendt M, Kittstein W, Furstenberger G, Marks F (1984). The mouse ear edema: a quantitatively evaluable assay for tumor promoting compounds and for inhibitors of tumor promotion. *Cancer Letters*.

[27] Aggarwal BB (2003). Signalling pathways of the TNF superfamily: a double-edged sword. *Nature Reviews Immunology*.

[28] Garcia-Diaz M, Bebenek K, Sabariegos R (2002). DNA polymerase *λ*, a novel DNA repair enzyme in human cells. *The Journal of Biological Chemistry*.

[29] Singhal RK, Wilson SH (1993). Short gap-filling synthesis by DNA polymerase *β* is processive. *Journal of Biological Chemistry*.

[30] Matsumoto Y, Kim K (1995). Excision of deoxyribose phosphate residues by DNA polymerase *β* during DNA repair. *Science*.

[31] Sobol RW, Horton JK, Kühn R (1996). Requirement of mammalian DNA polymerase-*β* in base-excision repair. *Nature*.

[32] Ramadan K, Shevelev IV, Maga G, Bscher UH (2002). DNA polymerase *λ* from calf thymus preferentially replicates damaged DNA. *Journal of Biological Chemistry*.

[33] Sugo N, Aratani Y, Nagashima Y, Kubota Y, Koyama H (2000). Neonatal lethality with abnormal neurogenesis in mice deficient in DNA polymerase *β*. *EMBO Journal*.

[34] García-Díaz M, Bebenek K, Kunkel TA, Blanco L (2001). Identification of an intrinsic 5′-deoxyribose-5-phosphate lyase activity in human DNA polymerase *λ*: a possible role in base excision repair. *Journal of Biological Chemistry*.

[35] Hirose F, Hotta Y, Yamaguchi M, Matsukage A (1989). Difference in the expression level of DNA polymerase *β* among mouse tissues: high expression in the pachytene spermatocyte. *Experimental Cell Research*.

[36] Garcia-Diaz M, Dominguez O, Lopez-Fernandez LA (2000). DNA polymerase *λ*, a novel DNA repair enzyme in human cells. *Journal of Molecular Biology*.

[37] Bertocci B, De Smet A, Flatter E (2002). Cutting edge: DNA polymerases *μ* and *λ* are dispensable for Ig gene hypermutation. *Journal of Immunology*.

[38] Tanaka H, Hasegawa T, Matsushita M, Miichi H, Hayashi S (1987). Quantitative evaluation of ocular anti-inflammatory drugs based on measurements of corneal temperature in rabbits: dexamethasone and glycyrrhizin. *Ophthalmic Research*.

[39] Ohuchi K, Kamada Y, Levine L, Tsurufuji S (1981). Glycyrrhizin inhibits prostaglandin E2 production by activated peritoneal macrophages from rats. *Prostaglandins and Medicine*.

[40] Ichikawa Y, Mizoguchi Y, Kioka K, Kobayashi K (1989). Effect of glycyrrhizin on the production of platelet-activating factor from rat peritoneal exudate cells. *Japanese Journal of Allergology*.

[41] Pompei R, Flore O, Marccialis MA, Pani A, Loddo B (1979). Glycyrrhizic acid inhibits virus growth and inactivates virus particles. *Nature*.

[42] Ito M, Nakashima H, Baba M (1987). Inhibitory effect of glycyrrhizin on the in vitro infectivity and cytopathic activity of the human immunodeficiency virus [HIV (HTLV-III/LAV)]. *Antiviral Research*.

[43] Abe N, Ebina T, Ishida N (1982). Interferon induction by glycyrrhizin and glycyrrhetinic acid in mice. *Microbiology and Immunology*.

[44] Finney RSH, Somers GF (1958). The antiinflammatory activity of glycyrrhetinic acid and derivatives. *Journal of Pharmacy and Pharmacology*.

[45] Capasso F, Mascolo N, Autore G, Duraccio MR (1983). Glycyrrhetinic acid, leucocytes and prostaglandins. *Journal of Pharmacy and Pharmacology*.

[46] Nishino H, Yoshioka K, Iwashima A (1986). Glycyrrhetic acid inhibits tumor-promoting activity of teleocidin and 12-*O*-tetradecanoylphorbol-13-acetate in two-stage mouse skin carcinogenesis. *Japanese Journal of Cancer Research*.

[47] Nose M, Ito M, Kamimura K, Shimizu M, Ogihara Y (1994). A comparison of the antihepatotoxic activity between glycyrrhizin and glycyrrhetinic acid. *Planta Medica*.

[48] Abe H, Ohya N, Yamamoto KF, Shibuya T, Arichi S, Odashima S (1987). Effects of glycyrrhizin and glycyrrhetinic acid on growth and melanogenesis in cultured B16 melanoma cells. *European Journal of Cancer and Clinical Oncology*.

[49] Monder C, Stewart PM, Lakshmi V, Valentino R, Burt D, Edwards CRW (1989). Licorice inhibits corticosteroid 11*β*-dehydrogenase of rat kidney and liver: in vivo and in vitro studies. *Endocrinology*.

[50] Medeiros R, Otuki MF, Avellar MCW, Calixto JB (2007). Mechanisms underlying the inhibitory actions of the pentacyclic triterpene *α*-amyrin in the mouse skin inflammation induced by phorbol ester 12-*O*-tetradecanoylphorbol-13-acetate. *European Journal of Pharmacology*.

